# Automated Protocol Suggestions for Cranial MRI Examinations Using Locally Fine-tuned BERT Models

**DOI:** 10.1007/s00062-025-01554-z

**Published:** 2025-08-18

**Authors:** Christian Boschenriedter, Christian Rubbert, Marius Vach, Julian Caspers

**Affiliations:** Department of Diagnostic and Interventional Radiology, Medical Faculty and University Hospital Düsseldorf, Heinrich-Heine-University Düsseldorf, Moorenstraße 5, 40225 Düsseldorf, Germany

**Keywords:** Neuroradiology, MRI, Protocol suggestion, NLP, BERT

## Abstract

Selection of appropriate imaging sequences protocols for cranial magnetic resonance imaging (MRI) is crucial to address the medical question and adequately support patient care. Inappropriate protocol selection can compromise diagnostic accuracy, extend scan duration, and increase the risk of misdiagnosis. Typically, radiologists determine scanning protocols based on their expertise, a process that can be time-consuming and subject to variability. Language models offer the potential to streamline this process. This study investigates the capability of bidirectional encoder representations from transformers (BERT)-based models to suggest appropriate MRI protocols based on referral information.

A total of 410 anonymized electronic referrals for cranial MRI from a local order-entry system were categorized into nine protocol classes by an experienced neuroradiologist. A locally hosted instance of four different, pre-trained BERT-based classifiers (BERT, ModernBERT, GottBERT, and medBERT.de) were trained to classify protocols based on referral entries, including preliminary diagnoses, prior treatment history, and clinical questions. Each model was additionally fine-tuned for local language on a large dataset of electronic referrals.

The model based on medBERT.de with local language fine-tuning was the best-performing model and correctly predicted 81% of all protocols, achieving a macro-F1 score of 0.71, macro-precision and macro-recall values of 0.73 and 0.71, respectively. Moreover, we were able to show that local language fine-tuning led to performance improvements across all models.

These results demonstrate the potential of language models to predict MRI protocols, even with limited training data. This approach could accelerate and standardize radiological protocol selection, offering significant benefits for clinical workflows.

## Introduction

Magnetic resonance imaging (MRI) is an essential diagnostic tool in current healthcare and belongs to the core elements of radiologic work. The efficacy of this modality relies heavily on selecting appropriate imaging sequences tailored to the patient’s medical context and the medical question that should be answered with this examination. Suboptimal protocol selection can lead to incomplete imaging, increased scan durations, and diagnostic inaccuracies. Conventionally, protocol determination is performed manually by radiologists, which is not only labor-intensive but also prone to interindividual variability and errors [6]. Ginat et al. analyzed MRI protocoling errors in the neuroradiology department across 4244 MRI exams over a six-month period and identified a total of 140 protocol-related issues [6]. A recent study by Denck et al. has found that through automated protocoling and workflow optimizations, 37% of these errors could be mitigated [3].

Recent advancements in artificial intelligence and natural language processing (NLP) have opened new avenues for optimizing healthcare workflows. In particular, large language models (LLMs) trained on medical texts show promise for automating tasks traditionally requiring expert input. Automated protocol suggestions have been explored only to a very limited extent in previous research [3]. In a study by Gertz et al., the publicly available GPT‑4 model was used to predict basic protocol parameters from a given radiology request form, including modality, body region, use of contrast agent, and, if applicable, the specific contrast phases [5]. Lee employed a convolutional neural network to classify musculoskeletal MRI indications into either a routine or a tumor-specific MRI protocol [8]. Huhtanen et al. utilized Finnish BERT, a language-fine-tuned encoder-only model based on transformer architecture, as well as GPT‑3.5 to classify clinical referral texts for emergency brain MRI protocoling with a total of twelve protocol classes [7]. However, more comprehensive approaches for automated MRI protocol recommendations remain largely unexplored.

Bidirectional encoder representations from transformers (BERT) marked a major breakthrough in NLP [4]. Its innovative architecture and training methodology have set new standards across multiple benchmarks, making it particularly effective for tasks requiring deep contextual understanding of language. Unlike traditional models that process text sequentially in one direction, BERT employs bidirectional contextual understanding, allowing it to analyze a word’s meaning by considering both the preceding and succeeding context. BERT’s development involves a two-step training process, consisting of pre-training and fine-tuning. During pre-training, the model is exposed to large text corpora to learn general language patterns and relationships. This phase includes tasks like masked language modeling, where random words are hidden and the model predicts them based on context, and next sentence prediction, which helps the model understand the logical relationships between sentences. After pre-training, BERT can be fine-tuned for specific tasks such as text classification or question answering. By leveraging its pre-learned knowledge, BERT adapts efficiently to domain-specific applications, even with limited additional training data.

This study aims to evaluate the capability of four different BERT models to assist in MRI protocol selection by analyzing referral entries and predicting appropriate MRI protocols. Furthermore, the influence of language-specific and domain-specific model-selection as well as the effect of local fine-tuning is analyzed. When proven feasible, such models have the potential to improve workflow efficiency and standardization in radiological practice.

## Materials and Methods

### Data Collection and Annotation

Due to the fully anonymized analysis of retrospective data, the requirement for written informed consent was waived by the ethics committee in accordance with local laws and regulations.

We retrospectively collected 692 anonymized electronic referrals for neuroradiological MRI examinations, written in German, from the local hospital’s order-entry system between January and November 2018. Each referral included entries for preliminary diagnoses, prior treatment history, and clinical questions, all of which were concatenated into a single text string composed of unstructured free text. This input sequences used for classification-head fine-tuning varied in length, with an average of 53 tokens (standard deviation: 31). The shortest input consisted of 13 tokens, while the longest input contained 169 tokens. All inputs were therefore well within the model’s maximum context window of (at least) 512 tokens, meaning no truncation or splitting of inputs was necessary during training. Additional patient information, such as age or sex, or data from health records was not collected. Each referral was assigned to one of nine predefined MRI protocol classes (“MRI brain with contrast”, “MRI brain without contrast”, “MRI stroke without perfusion”, “MRI cerebellopontine angle”, “MRI multiple sclerosis”, “MRI movement disorder”, “MRI stroke with perfusion”, “MRI pituitary gland”, and “MRI epilepsy”) by a senior neuroradiologist with 12 years of experience, who labeled the cases independently of the MRI protocol that was ultimately performed. This ensured high-quality and unbiased annotations, but naturally limited the volume of data we could include. These definitions served as the ground truth for model training and evaluation. We excluded non-brain examinations and retained only those featuring the most commonly used MRI protocols. This resulted in a total of 410 remaining MRI requests.

### Model Training and Evaluation

For the prediction of MRI protocols, we implemented classifiers for multiclass classification based on BERT models. We tested the base versions of four BERT-based models (“google-bert/bert-base-uncased”, “TUM/GottBERT_base_last”, “answerdotai/ModernBERT-base”, and “GerMedBERT/medbert-512”) including language- and domain-specific pretrained implementations to determine which one is best suited for predicting the correct protocol class after training on our labeled datasets. BERT is pretrained on a large corpus of English text in a self-supervised manner using masked language modeling and next sentence prediction to learn deep bidirectional representations [4]. ModernBERT enhances BERT’s efficiency by incorporating architectural refinements and optimizations tailored to contemporary hardware and NLP challenges [10]. It aims to achieve a better trade-off between model complexity and performance, making it more suitable for large-scale applications. GottBERT is a language-specific BERT implementations for the German language [9]. Unlike general-purpose BERT models, this model is optimized for linguistic nuances and domain-specific vocabulary in German. medBERT.de is specifically fine-tuned on a large dataset of German medical texts, clinical notes, research papers, and healthcare-related documents [2].

To create the BERT-based classifiers, a sequence classification head for a nine-class classification task was added to each BERT model. We then trained the classifier head to predict the MRI protocol labels using stochastic gradient descent and 5‑fold cross-validation. The weights of the BERT encoder were frozen.

For model evaluation, we conducted a stratified 5‑fold cross-validation based on the protocol classes. To prevent overfitting during training, we employed early stopping by selecting the model checkpoint corresponding to the epoch with the lowest mean validation loss, which occurred within a total of 22 training epochs. Addressing the issue of class imbalance in a multi-class classification setting, we employed a weighted cross-entropy loss function. The class-specific weights were computed based on the inverse frequency of each class within the dataset, ensuring that underrepresented classes contribute more significantly to the loss function. This approach ensures that the model does not disproportionately favor the most frequent classes, thereby enhancing its ability to learn meaningful representations across all categories. In order to minimize the loss function, we used the Adam optimization algorithm with a learning rate of 0.00005. A batch size of 32 was used, and training samples were shuffled at each epoch. The training code was implemented in PyTorch (version 2.3.1) and all training was conducted on an NVIDIA Titan V GPU with 12 GB of VRAM. The total time required for training and evaluating medBERT.de as base model with prior local language fine-tuning across all five cross-validation folds was 5 min and 50 s. The average inference time for predicting a single protocol was 0.1 s.

### Local Language Fine-tuning

**Fig. 1:**
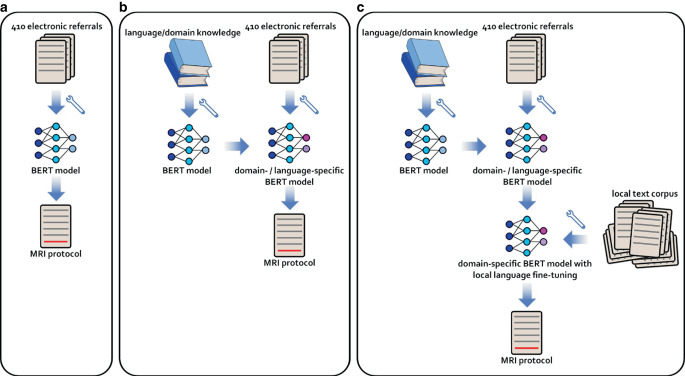
Workflow overview. **a** Initial protocol prediction using a standard BERT model. **b** Enhancement of the model through language- and domain-specific pretraining. **c** Further refinement via local language fine-tuning of the language- and domain-adapted BERT model

In addition to the classifier based on the respective base-models, the effect of local language fine-tuning of the BERT encoder model was evaluated (Fig. 1). For this, each BERT model was fine-tuned with a masked language modelling task on a large dataset of 123,274 anonymized electronic referrals. This dataset encompassed MRI requests from all body parts of the local hospital between January 2018 and November 2022, including but not limited to cranial MRI. The fine-tuning process was conducted over 5 epochs with a learning rate of 0.00002 and a weight decay of 0.01. We applied gradient accumulation with an effective batch size of 32 and early stopping by selecting the model checkpoint from the epoch with the lowest average validation loss. The fine-tuned models were then trained and evaluated for MRI protocol classification in the same way as the base-models using 5‑fold cross validation on the 410 labeled referrals.

### Statistical Analysis

To evaluate model performance comprehensively, we employed multiple metrics, including accuracy, balanced accuracy, F1-score, precision and recall. All metrics were averaged over the 5 folds of cross-validation. Macro-averaging was selected for calculation of precision, recall and F1-scores to emphasize the performance on minority classes by averaging these scores across all classes without weighting by class frequency. Additionally, confusion matrices were analyzed to gain deeper insights into misclassification patterns and model biases.

We conducted five independent runs of model evaluation to obtain robust performance estimates. This allowed us to compute 95% Bayesian confidence intervals (CI) for all metrics, ensuring statistical reliability. The evaluation and statistical analysis were implemented in Python 3.10.12 using the scipy (version 1.8.0) and sklearn (version 1.4.2) libraries.

The code for model training as well as model evaluation and analysis can be found at github.com/deepneuroimaging/bert4mri.

## Results

### Sample Characteristics

**Fig. 2:**
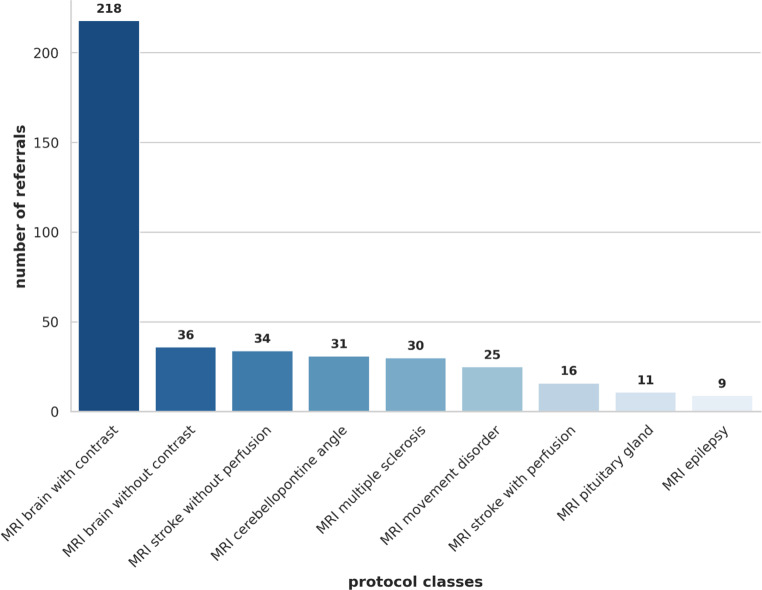
Distribution of protocol classes

A total of 410 referrals for cranial MRI including preliminary diagnoses, prior treatment history, and clinical questions were included in the study. Those were assigned to nine protocol classes (Fig. 2), including “MRI brain with contrast” (*n* = 218), “MRI brain without contrast” (*n* = 36), “MRI stroke without perfusion” (*n* = 34), “MRI cerebellopontine angle” (*n* = 31), “MRI multiple sclerosis” (*n* = 30), “MRI movement disorder” (*n* = 25), “MRI stroke with perfusion” (*n* = 16), “MRI pituitary gland” (*n* = 11), and “MRI epilepsy” (*n* = 9).

### Model Evaluation

**Fig. 3:**
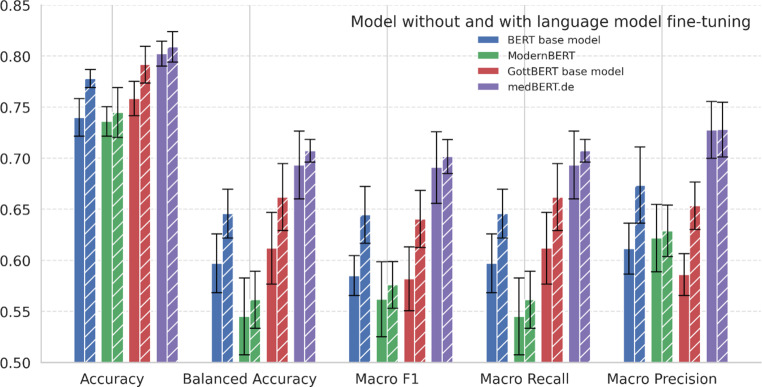
Comparison of classification performance across all models before (solid) and after (hatched) local language model fine-tuning. Error bars represent 95% Bayesian confidence intervals

All metrics for each model, both before and after local language fine-tuning, are presented in Table 1 and Fig. 3. The classifier model based on medBERT.de provided the best overall performance (accuracy: 0.80; F1: 0.69), followed by the classifiers based on GottBERT (accuracy: 0.76, F1: 0.58), the BERT base model (accuracy: 0.74; F1: 0.59), and ModernBERT (accuracy: 0.74; F1: 0.56).

**Table 1:** Comparison of all models before and after language model fine-tuning. Values in parentheses represent 95% confidence intervals. In single-label multiclass classification, micro precision, micro recall, and micro F1 score yield identical values. In comparison, a dummy classifier achieves evaluation metrics of 0.11, reflecting random performance across the nine classes.

	Accuracy	Balanced Accuracy	Macro F1	Macro Recall	Macro Precision	Micro Recall/Precision/F1
BERT base model	0.74 (0.72, 0.76)	0.60 (0.57, 0.63)	0.59 (0.57, 0.60)	0.60 (0.57, 0.63)	0.61 (0.59, 0.64)	0.74 (0.72, 0.76)
BERT base model + language fine-tuning	0.78 (0.77, 0.79)	0.65 (0.62, 0.67)	0.64 (0.62, 0.67)	0.65 (0.62, 0.67)	0.67 (0.64, 0.71)	0.78 (0.77, 0.79)
ModernBERT	0.74 (0.72, 0.75)	0.55 (0.51, 0.58)	0.56 (0.53, 0.60)	0.55 (0.51, 0.58)	0.62 (0.59, 0.65)	0.74 (0.72, 0.75)
ModernBERT + language fine-tuning	0.76 (0.74, 0.78)	0.56 (0.53, 0.59)	0.58 (0.55, 0.61)	0.61 (0.58, 0.65)	0.59 (0.57, 0.61)	0.74 (0.72, 0.77)
GottBERT base model	0.76 (0.74, 0.78)	0.61 (0.58, 0.65)	0.58 (0.55, 0.61)	0.61 (0.58, 0.65)	0.59 (0.57, 0.61)	0.76 (0.74, 0.78)
GottBERT base model + language fine-tuning	0.79 (0.77, 0.81)	0.66 (0.63, 0.69)	0.64 (0.61, 0.67)	0.66 (0.63, 0.69)	0.65 (0.63, 0.68)	0.79 (0.77, 0.81)
medBERT.de	0.80 (0.79, 0.81)	0.69 (0.66, 0.73)	0.69 (0.66, 0.73)	0.69 (0.66, 0.73)	0.73 (0.70, 0.76)	0.80 (0.79, 0.81)
medBERT.de + language fine-tuning	0.81 (0.79, 0.82)	0.71 (0.70, 0.72)	0.71 (0.69, 0.72)	0.71 (0.70, 0.72)	0.73 (0.70, 0.76)	0.81 (0.79, 0.82)

After local language fine-tuning, all models showed improved performance, with the classifier based on medBERT.de achieving the highest overall performance (accuracy: 0.81; F1: 0.71). Our results further demonstrate that language fine-tuning significantly enhanced the performance of the BERT base model with respect to both accuracy and F1 score, while the improvements observed in the other models were comparatively modest.

**Fig. 4:**
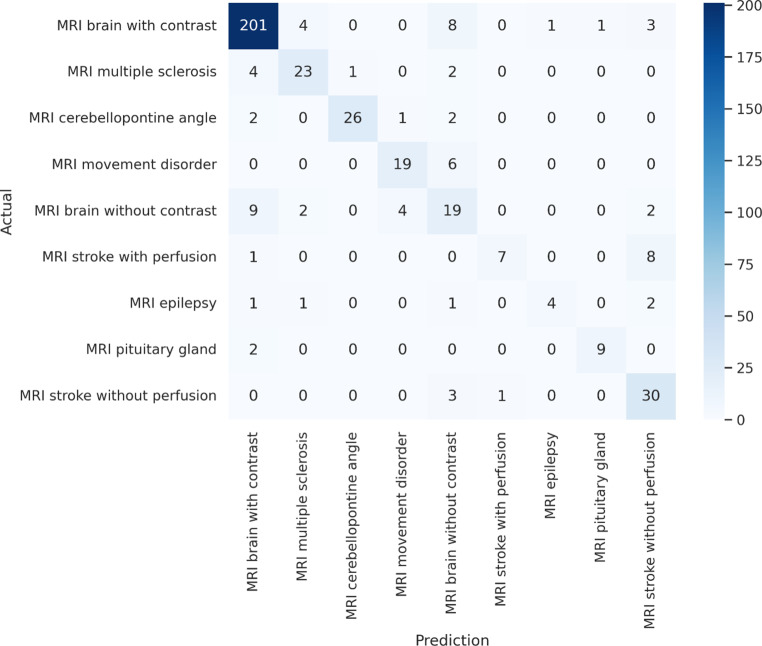
Confusion matrix for the most accurate model (medBERT.de as base model after prior local language fine-tuning)

The confusion matrix illustrates the performance of the most accurate classifier model (medBERT.de with prior local language fine-tuning) across all categories (Fig. 4). In the per-class (one-vs-rest) evaluation, the most frequent protocol class, “MRI brain with contrast”, was predicted with a precision of 0.89 (95% CI: 0.86, 0.91) and recall of 0.91 (95% CI: 0.90, 0.93). In contrast, the least frequent class, “MRI epilepsy”, achieved a precision of 0.61 (95% CI: 0.54, 0.69) and a recall of 0.53 (95% CI: 0.47, 0.60). The corresponding metrics for these and the remaining protocol classes are summarized in Table 2.

**Table 2:** Per-class (one-vs-rest) evaluation metrics of the model based on medBERT.de after local language fine-tuning. Values in parentheses represent 95% confidence intervals.

	Precision	Recall
MRI brain with contrast	0.89 (0.86, 0.91)	0.91 (0.90, 0.93)
MRI brain without contrast	0.55 (0.51, 0.60)	0.51 (0.48, 0.53)
MRI stroke without perfusion	0.68 (0.65, 0.71)	0.76 (0.63, 0.87)
MRI cerebellopontine angle	0.88 (0.86, 0.90)	0.84 (0.84, 0.84)
MRI multiple sclerosis	0.82 (0.72, 0.91)	0.79 (0.75, 0.82)
MRI movement disorder	0.79 (0.74, 0.85)	0.74 (0.66, 0.81)
MRI stroke with perfusion	0.66 (0.53, 0.79)	0.45 (0.33, 0.57)
MRI pituitary gland	0.87 (0.82, 0.93)	0.78 (0.72, 0.84)
MRI epilepsy	0.61 (0.54, 0.69)	0.53 (0.47, 0.60)

## Discussion

In this study, the feasibility of predicting MRI protocols based on the electronic referral texts with different BERT-based models was evaluated. Our results demonstrate the potential of language models for automating protocol selection in cranial MRI and suggest their applicability to other domains, such as non-neuroradiological MRI or CT protocol prediction. While our most accurate model (medBERT.de as base model with prior local language fine-tuning) achieved high overall performance, its accuracy varied between protocol classes, reflecting the challenges posed by class imbalance. As expected, the most frequent class was predicted with high accuracy, whereas rare classes were often misclassified. Despite these limitations, the model’s robust F1 score suggests strong discriminative ability, even with limited training data.

Selective automation may offer a promising strategy for practical deployment, wherein our most accurate model autonomously assigns only the high-confidence cases of the class “MRI brain with contrast”, while all other cases are referred for manual review. This approach aims to leverage the model’s strengths in distinguishing this specific class with high precision. As an initial evaluation of its feasibility, we calculated precision and recall for the class “MRI brain with contrast” versus all other classes, yielding values of 0.89 and 0.91, respectively. While these results are encouraging, they remain insufficient for reliable clinical implementation at this stage.

medBERT.de proved to be the best base model for classifying German-language referrals. This can be attributed to its domain-specific pretraining on medical texts, which likely provided a more suitable linguistic and contextual representation for the task. In contrast, BERT, ModernBERT, and GottBERT exhibited substantial lower performances with no relevant differences between these models. One possible reason is that standard BERT lacks domain-specific adaptation, making it less effective for medical terminology. Similarly, while GottBERT is trained on a large corpus of German texts, it does not focus on medical language, which may explain its limited suitability for this classification task.

Local language fine-tuning is hypothesized to provide significant benefits primarily due to the incorporation of both linguistic and domain-specific contexts that were previously absent [11]. It yields the most significant performance improvements for BERT, ModernBERT, and GottBERT, bringing them closer to the performance of medBERT.de. However, while the relative gain from fine-tuning is smaller for medBERT.de, it still consistently outperforms all other models. For non-German and non-domain-specific models, this advantage is evident as the fine-tuning introduces the necessary language and domain context. In the case of medBERT.de, although the benefits are less immediately apparent, they can likely be attributed to the model learning highly specialized terminology, including specific abbreviations and contextual usage unique to the local medical domain, which significantly improves its performance in this context. The positive effect of hospital-specific domain adaptation in BERT-based models was also demonstrated in a study by Agarwal et al., who utilized these models for classifying neuroradiology reports [1]. This highlights the effectiveness of combining a language- and domain-adapted pretraining approach with local language fine-tuning, making it the optimal strategy for domain-specific NLP tasks.

A key advantage of the BERT-based models employed in this study is their ability to run locally, even on standard computers with moderate processing power. This ensures that potentially sensitive patient information from electronic referrals does not need to be shared with external data centers outside the clinic, thereby enhancing data privacy. Additionally, while fine-tuning is generally possible for many language models, it is often more straightforward to implement for locally deployed models, allowing them to better adapt to clinic-specific jargon. In this regard, relying on a domain- and language-specific pre-trained and pre-tuned model like medBERT.de significantly reduces the amount of in-house training required on the clinic’s own electronic referrals. This not only accelerates model adaptation but also conserves computational resources, making the approach more efficient and cost-effective.

Despite the promising results, this study has several limitations that should be considered. Our dataset exhibits a significant class imbalance, with the majority of cases consisting of follow-up examinations after tumor resections, as well as monitoring of aneurysms and multiple sclerosis lesions. This imbalance poses a challenge for model training, as it may lead to suboptimal performance in underrepresented classes. To improve future model performance, it is essential to collect training data that includes a broader range of less frequent clinical questions to ensure that the model generalizes well across diverse clinical scenarios.

While the sample size is limited, cross-validation maximizes the effective use of available data and helps mitigate potential overfitting. Nevertheless, future studies with larger datasets will be necessary to further validate and generalize the findings.

The integration of language models into radiological workflows holds significant potential for improving efficiency by reducing the time required for protocol selection and enhancing the overall standardization of imaging procedures. By automating and optimizing these processes, such models could help radiologists select the most appropriate protocols faster, ensuring that imaging studies are consistent and of high quality. This could lead to more streamlined workflows, reduced human error, and potentially better patient outcomes.
